# Botulinum protease-cleaved SNARE fragments induce cytotoxicity in neuroblastoma cells

**DOI:** 10.1111/jnc.12645

**Published:** 2014-01-23

**Authors:** Jason Arsenault, Sabine A G Cuijpers, Enrico Ferrari, Dhevahi Niranjan, Aleksander Rust, Charlotte Leese, John A O'Brien, Thomas Binz, Bazbek Davletov

**Affiliations:** * MRC Laboratory of Molecular BiologyCambridge, UK; † University of Lincoln, School of Life SciencesLincoln, UK; ‡ Department of Biomedical Science, University of SheffieldSheffield, UK; § Medizinische Hochschule Hannover, Institut für BiochemieHannover, Germany

**Keywords:** botulinum, cytotoxicity, neuro2A, SNARE, syntaxin, transfection reagents

## Abstract

Soluble *N*-ethylmaleimide sensitive factor attachment protein receptors (SNAREs) are crucial for exocytosis, trafficking, and neurite outgrowth, where vesicular SNAREs are directed toward their partner target SNAREs: synaptosomal-associated protein of 25 kDa and syntaxin. SNARE proteins are normally membrane bound, but can be cleaved and released by botulinum neurotoxins. We found that botulinum proteases types C and D can easily be transduced into endocrine cells using DNA-transfection reagents. Following administration of the C and D proteases into normally refractory Neuro2A neuroblastoma cells, the SNARE proteins were cleaved with high efficiency within hours. Remarkably, botulinum protease exposures led to cytotoxicity evidenced by spectrophotometric assays and propidium iodide penetration into the nuclei. Direct delivery of SNARE fragments into the neuroblastoma cells reduced viability similar to botulinum proteases' application. We observed synergistic cytotoxic effects of the botulinum proteases, which may be explained by the release and interaction of soluble SNARE fragments. We show for the first time that previously observed cytotoxicity of botulinum neurotoxins/C in neurons could be achieved in cells of neuroendocrine origin with implications for medical uses of botulinum preparations.

Vesicular transport, protein trafficking, and exocytosis necessitate membrane fusion of vesicles, which is catalyzed by soluble *N*-ethylmaleimide sensitive factor attachment protein receptors (SNAREs) (Bock *et al*. 2001; Jahn *et al*. 2003; Meunier *et al*. 2003; Bajohrs *et al*. 2005; Sudhof and Rothman 2009; Mohrmann *et al*. 2010; Gao *et al*. 2012; Risselada and Grubmuller 2012). SNAREs are divided into two categories depending on their cellular locations: v-SNAREs on vesicles (also known as synaptobrevins; or vesicle-associated membrane proteins) and the t-SNAREs located on the target membrane, the synaptosome-associated protein of 25 kDa (SNAP25), and syntaxin (Bajohrs *et al*. 2005). SNAREs form a highly stable, tetrahelical coil–coil bundle that fuses the vesicle to the target membrane (Fig. 1a). Syntaxin and synaptobrevin each contribute one α-helix, whereas the SNAP25 protein contributes two (Wendler and Tooze 2001; Sudhof and Rothman 2009). There have been over 35 different SNARE isoforms identified, each associated with distinct organelles that participate in the precise and scrupulous vesicle fusion essential for proper cellular function (Bock *et al*. 2001; Wendler and Tooze 2001; Koticha *et al*. 2002; Xu and Xu 2008).

**Fig. 1:**
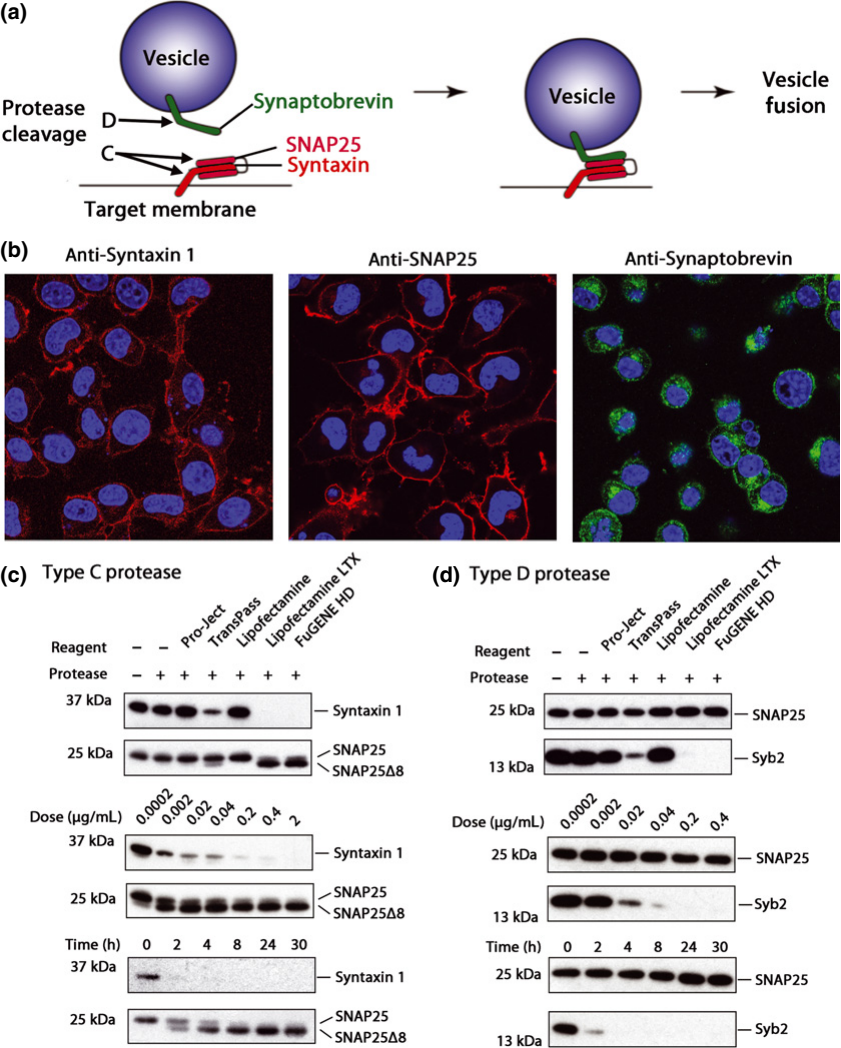
Efficient transduction of botulinum type C and D proteases into neuroblastoma cells. (a) Schematic of the complex formation by synaptobrevin, v-soluble *N*-ethylmaleimide-sensitive factor attachment protein receptor (SNARE) (green) and syntaxin/SNAP25, t-SNAREs (red and fuchsia) necessary for vesicle fusion with the cell membrane. The substrates of the botulinum protease type C are syntaxin and SNAP25, whereas the type D protease cleaves only synaptobrevin. (b) Confocal image showing syntaxin 1 (red; left), SNAP25 (red; middle), and synaptobrevins 1-3 (green; right) in Neuro2A cells as revealed by immunocytochemistry. (c) Comparison of the efficiency of different reagents used to deliver botulinum protease type C (top panel). Cleavage of syntaxins evidenced by the disappearance of the immunoreactive band, whereas cleavage of SNAP25 is evidenced by the appearance of a proteolytic product SNAP25Δ8. SNAP25 immunoreactivity served as a loading control. Middle panel shows the dose dependence of SNARE cleavage following 20 h treatment. Bottom panel shows a time course using 0.2 μg/mL of the type C protease. (d) Comparison of different reagents used to deliver botulinum protease type D (top panel) and efficacy (lower panels) of synaptobrevin 2 (Syb2) cleavage evidenced by the disappearance of the immunoreactive band. SNAP25 was used as a loading control.

Botulinum neurotoxins (BoNT) have been invaluable in the studies of SNARE proteins and exocytosis (Erbguth and Naumann 1999; Sorensen *et al*. 2002). BoNTs are natively produced by anaerobic bacteria of the genus *Clostridium* and are responsible for the deadly disease called botulism manifested by neuromuscular paralysis (Erbguth and Naumann 1999; Montecucco and Molgo 2005). The BoNTs carry selective SNARE proteases, which have highly precise substrate recognition sites (Montecucco and Schiavo 1994; Montal 2010). BoNTs have been utilized in many medical and biotechnological applications (Davletov *et al*. 2005; Chaddock and Marks 2006; Foster *et al*. 2006; Ferrari *et al*. 2011; Arsenault *et al*. 2013). A typical BoNT is expressed as a single-chain precursor protein that is processed into two polypeptide chains. The SNARE protease (50 kDa light chain) located at the N terminus is linked, via a disulfide bond, to the C terminal part (100 kDa heavy chain) which is composed of the translocation domain and the receptor-binding domain (Chaddock and Marks 2006; Binz and Rummel 2009). Among seven commonly known BoNT serotypes (A–G) BoNT/A, C, and E cleave SNAP25, BoNT/B, D, F, and G cleave synaptobrevins, and BoNT/C also cleaves syntaxin (Montecucco and Schiavo 1994; Rummel *et al*. 2004; Dong *et al*. 2006; Antonucci *et al*. 2008; Binz *et al*. 2010). To reach their intraneuronal substrates, BoNTs first bind neuronal surface gangliosides and then a synaptic vesicle protein on the pre-synaptic membrane for subsequent internalization (Montecucco *et al*. 1994; Rummel *et al*. 2004, 2009; Dong *et al*. 2006; Mahrhold *et al*. 2006; Binz and Rummel 2009). Once the internalized vesicle acidifies, the translocation domain forms a putative protein transduction channel that enables translocation of the protease into the cytosol following reduction in its disulfide bond (Koriazova and Montal 2003; Puhar *et al*. 2004; Fischer 2013; Pirazzini *et al*. 2013). BoNTs are potentially lethal as they paralyze muscles, such as the diaphragm, but are thought to be non-cytotoxic to neurons with the clear exception of BoNT/C and possibly BoNT/E (Williamson and Neale 1998; Berliocchi *et al*. 2005; Zhao *et al*. 2010; Peng *et al*. 2013). The underlying causes of BoNT/C-mediated neurotoxicity are still under investigation. BoNT/C-released syntaxin fragment (aa 1-253) may cause cytotoxicity, but was thought to be rapidly degraded as observed by the loss of syntaxin immunoreactivity (Foran *et al*. 2003). However, immunoreactivity is not necessarily a measure of protein presence and other studies have been able to identify syntaxin fragments in cell extracts (Tsukamoto *et al*. 2012). If these fragments are free to diffuse and to engage SNAP25 and synaptobrevin or other non-neuronal SNAREs (Fasshauer *et al*. 1999), then they may potentially interfere with the scrupulous fusion events. Others have reported that BoNT/C-affected neurons first undergo axonal degeneration, possibly because of trafficking problems, followed by apoptosis (Berliocchi *et al*. 2005; Zhao *et al*. 2010; Peng *et al*. 2013).

BoNT protease-based medicines have recently been proposed for the treatment of endocrine disorders and therefore we investigated the possible cytotoxic effects of SNARE cleavage in endocrine cells. We have utilized model neuroblastoma cells (N2A) that abundantly express all three neuronal SNARE proteins: syntaxin 1, SNAP25, and synaptobrevin 2 (Syb2). N2A cells have been shown to be resistant to native BoNTs because of lack of neuronal gangliosides (Yowler *et al*. 2002). We thus used transfection reagents to internalize the proteases into the cell interior as it was shown before that polycationic lipid and polymers can enable the endocytosis and translocation of the BoNT type A protease as well as other peptides and proteins into cells (Kuo *et al*. 2010; Oba and Tanaka 2012). We now show that a selection of transfection reagents can mediate the intracellular entry of the botulinum protease serotypes C and D into neuroblastoma cells allowing investigation of botulinum action. Our results show that the intracellular cleavage of syntaxin and SNAP25 by the type C protease and synaptobrevin by the type D protease can cause severe cytotoxic effects. The soluble SNARE fragments were able to form ternary complexes and when transduced, triggered loss of cell viability mimicking the type C and D protease effects.

## Materials and methods

### Cell culture

Mouse Neuro2A cells (ATCC: CCL-131; LGC Standards; Teddington, UK) were grown in a 37°C incubator at 5% CO_2_ in low-glucose Dulbecco's modified eagle medium (Gibco; Paisly, UK) supplemented with 10% fetal clone 1 calf serum (HyClone; Fisher Scientific; Loughborough, UK) and 1% penicillin/streptomycin (P/S) (Invitrogen; Paisley, UK). Every 3–4 days, cells were washed with phosphate-buffered saline (PBS) and resuspended in culture medium using flow pressure, then counted by hemacytometer. Min6 and SH-SY5Y cells were cultured as previously described (Arsenault *et al*. 2013). Rat cortical neuronal cells were isolated as previously described (Arsenault *et al*. 2013). Sprague–Dawley rats of both sexes were obtained from the Medical Research Council Laboratory of Molecular Biology's Biological Services Group. Experiments were approved by the Medical Research Council Laboratory of Molecular Biology, Cambridge. Hela cells were cultured in high-glucose Dulbecco's modified eagle medium with 10% fetal clone 1 calf serum and 1% P/S. Cells were plated at 1 × 10^6^ cells per 9 cm culture dish (BD Biosciences; San Jose, CA, USA), at 8 × 10^4^ cells per well in uncoated 24-well plates (BD Biosciences) with or without cover slips, or at 8 × 10^3^ cells per well in uncoated 96-well plates (BD Bioscience).

### Protein and peptide synthesis

BoNT/A1 (1-872), rat SNAP25B (22-206) (all four Cys mutated to Ala), and rat synaptobrevin 2 (25-84) were prepared as Glutathione *S*-transferase (GST)-tagged proteins cleavable by thrombin and purified as previously described (Darios *et al*. 2010; Ferrari *et al*. 2012). Syntaxin 1 (1-226) was obtained from ATgen (Bio Trend, Köln, Germany). The production of serotype C and D proteases was described elsewhere (Vaidyanathan *et al*. 1999; Sikorra *et al*. 2006). Rat synaptobrevin 2 (25-52) Ac-RLQQTQAQVDEVVDIMRVNVDKVLERD-NH_2_ and syntaxin 1A (201-245) Ac-EIIKLENSIRELHDMFMDMAMLVESQGEMIDRIEYNVEHAVDYVE-NH_2_ peptides were prepared as previously described (Darios *et al*. 2010; Ferrari *et al*. 2012). Rat syntaxin 1A conjugated to Fluorescein isothiocyanate (FITC) FITC-Ahx-EIIKLENSIRELHDMFMDMAMLVESQGEMIDRIEYNVEHAVDYVE-NH_2_, rat complexin (31-59) Ac-GGGERKAKYAKMEAEREVMRQGIRDKYGIKKG-NH_2_, rat synaptobrevin 2 conjugated to FITC (31-55) sequence FITC-Ahx-RLQQTQAQVDEVVDIMRVNVDKVLE-NH_2_ and penetratin Ac-RQIKIWFQNRRMKWKK-NH_2_ were synthesized by Peptide Synthetics (Southampton, UK). Protein and peptide concentration was determined by Pierce bicinchoninic acid (BCA) protein assay kit (Thermo-scientific; Loughborough, UK) according to manufacturer protocol.

### Protein transduction

Transduction was performed 24 h after plating with proteins and peptides at desired concentrations that were pre-incubated for 30 min in 100 μL of Opti-MEM (GIBCO) with 2.5 μL of transfection reagents (per 500 μL of culture medium in a 24-well plate). Lipofectamine (Invitrogen), Lipofectamine LTX (Invitrogen), Transpass P (New England BioLabs; Hitchin, UK), and Fugene HD (Promega; Southampton, UK) were used as received from manufacturer. Pro-Ject (Thermo Scientific) was prepared according to manufacturer protocol. Cells were incubated with proteins and transfection reagents at 37°C in cell culture incubator for 42 h or as otherwise indicated.

### Confocal microscopy

Cells were grown on cover slips in 24-well plates and treated with compounds 16 h before fixation. Wells were washed once with PBS, and incubated in 4% paraformaldehyde (Alfa Aesar; Haysham, UK) in PBS for 20 min at 22°C. Wells were washed three times with PBS for 5 min and incubated 10 min in 10 mM NH_4_Cl (Sigma-Aldrich; Dorset, UK). The cells were then incubated for 30 min in a permeabilization solution composed of 0.1% Triton X-100 (Sigma-Aldrich) and 5% Bovine serum albumin (BSA; Sigma-Aldrich) in PBS at 22°C. Permeabilization solution was removed and replaced with 5% BSA in PBS containing appropriate primary antibodies for 90 min at 22°C. Mouse monoclonal anti-SNAP25 (SMI81; Novagen; EMD Millipore; Feltham, UK), rabbit polyclonal anti-synaptobrevin 1/2/3 antibody (Synaptic Systems; Goettingen, Germany), and mouse monoclonal anti-syntaxin 1 (clone HPC-1; Sigma-Aldrich), were diluted at 1 : 500. The wells were washed three times in PBS, then incubated for 30 min with Alexa Fluor® 594 goat anti-mouse IgG (H+L) and/or Alexa Fluor® 488 goat anti-rabbit IgG (H+L) (Invitrogen) diluted 1 : 800 in 5% BSA in PBS at 22°C. Wells were washed three times in PBS. Cover slips were overturned onto Vectashield (Vectorlabs; Orton Southgate, UK) mounting medium, sealed with nail varnish, and visualized on Zeiss 710 (Cambridge, UK) on 10 or 63X. The fluorescent gains intensities and pinhole size (1 AU) were identical between experimental samples.

### Western immunoblotting

The medium was removed from the wells and cells were incubated for 5 min in 100 μL loading buffer [56 mM sodium dodecyl sulfate (Sigma-Aldrich), 0.05 M Tris-HCl (Bio-Rad; Hemel Hempstead, UK) pH 6.8, 1.6 mM UltraPure EDTA (Gibco), 6.25% glycerol (Fisher Scientific), and 0.00001% bromophenol blue (Fisher Scientific)]. One unit of benzonase (Novagen; EMD Millipore) supplemented with 1 μL of 1 M MgCl_2_ was added to each well and plates were shaken at 1500 rpm for an additional 10 min. Samples were boiled for 1 min at 95°C then run on 12% Bis-Tris sodium dodecyl sulfate–polyacrylamide gel electrophoresis (SDS–PAGE) gels (Invitrogen). Migrated samples were transferred on Immobilon-P polyvinylidene difluoride membranes (EMD Millipore), and then incubated for 30 min in blotting solution (5% milk, 0.1% TWEEN 20 (Thermo Scientific) in PBS). All primary antibodies, including rabbit polyclonal anti-syntaxin 1b (Synaptic Systems), anti-syntaxin 1 (HPC-1, Sigma-Aldrich), and anti-synaptobrevin 2 clone 69.1 (Synaptic Systems), were diluted to 1 : 3000 in blotting solution and incubated for 1 h at 22°C. Membranes were washed three times in 0.1% TWEEN 20 in PBS for 5 min and then incubated for 30 min in blotting solution with secondary stabilized peroxidase-conjugated goat anti-mouse and/or anti-rabbit (Thermo Scientific) at 22°C. Membranes were washed three times for 5 min in 0.1% TWEEN 20 in PBS. Bands were illuminated using SuperSignal West Dura Extended Duration Substrate (Thermo Scientific) and signal was visualized by autoradiogram using Fuji Medical X-Ray films (Ross-on-wye, UK).

### Cell viability assays

Cells plated into 96-well plates (Costar; Sigma-Aldrich) exposed to 1/10 of the amount of the transduction recipe indicated above were incubated for 40 h. Cell Counting Kit-8 (CCK-8; Sigma-Aldrich) was used to determine cell survival (Ishiyama *et al*. 1997). Assay was performed according to manufacturer's protocol. Briefly, 10 μL of CCK-8 solution was added to each well and cells were incubated a further 2 h at 37°C. A Tecan Safire microplate reader (Männerdorf, Switzerland) was used to read absorbance at 450 nm in each well. All readings were normalized to untreated control conditions or as otherwise indicated. Cell-free medium with CCK-8 solution was used as a blank. BCA assay kit (Thermo-Scientific, Cramlington, UK) was used to monitor total protein content in each well. The medium was removed and adherent cells were gently washed once with PBS. BCA reagent was then added to each well-containing adherent cells, incubated for 1 h at 37°C and the total protein content was measured at 562 nm according to the manufacturer's protocol.

### Flow cytometry

Cells treated with compounds and transfection reagents were washed twice with PBS and resuspended in 10 mM HEPES (Fisher Scientific), 140 mM NaCl, 2.5 mM CaCl_2_, pH 7.4 with 2 μg/mL propidium iodide (PI; Sigma-Aldrich) into 12 × 75 mm round bottom test tube (Scientific Laboratory Supplies; Wilford, UK). After 10 min of incubation with PI, fluorescent intensities of the cell populations were measured using FACScalibur 2 (BD Bioscience). Proper gating was determined by untreated control, and laser intensity was determined by PI- and PI+ controls during each experiment. Data were interpreted using FlowJo version 9.4.4 (Tree Star Inc.; Ashland, USA).

### SNARE complex reactions

GST-synaptobrevin 2 (25-84) was incubated with or without the type D protease for 6 h at 22°C. Glutathione Sepharose 4B beads (GE Healthcare; Little Chalfont, UK) were added and incubated overnight at 4**°**C under constant rotation. Samples were washed four times with buffer A (0.8% w/v n-octyl-β-d-glucopyranoside (Sigma-Aldrich), 100 mM NaCl, 20 mM HEPES, pH 7.4) and divided into two tubes. A 1 : 1 ratio of syntaxin 1A (201-245) and SNAP25B (22-206) was added to one sample while syntaxin and buffer A were added to the other and incubated for 3 h at 22°C. Samples were washed four times with buffer A then heated at 95°C for 1 min and loaded on 10% Bis-Tris SDS–PAGE gel (Invitrogen). For the second assay GST-SNAP25 (22-206) was added to Glutathione Sepharose 4B beads (GE Healthcare) and incubated for 90 min at 4°C under constant rotation. Samples were washed four times with buffer A (0.8% w/v n-octyl-β-d-glucopyranoside (Sigma-Aldrich), 100 mM NaCl, 20 mM HEPES, pH 7.4) and divided into four tubes. Buffer A was added to the first sample, FITC-synaptobrevin (31-55) was added to the second sample. A 1 : 1 ratio of FITC-syntaxin 1A (201-245) and FITC-synaptobrevin (31-55) was added to the third sample and a 1 : 1 ratio of syntaxin 1A (1-226) and FITC-synaptobrevin (31-55) was added to the fourth sample. Samples were incubated for 30 min at 22°C then washed three times with buffer A, heated at 95°C for 1 min, and loaded on 10% Bis-Tris SDS–PAGE gel (Invitrogen). ChemiDoc XRS (Bio-Rad) was used to capture fluorescent and Coomassie images of the gels.

### Statistical analysis

All experiments were performed in at least three independent experiments. Results are presented as mean ± standard deviation (SD). Data analysis was performed using Graphpad Prism 5.0 (La Jolla, CA, USA). The unpaired two-tailed Student's *t*-test was used for comparison. A *p* < 0.05 was considered statistically significant.

## Results

### Direct delivery of botulinum proteases into neuroblastoma cells

First, we ascertained the presence of the SNARE proteins syntaxin, SNAP25, and synaptobrevin in mouse neuroblastoma N2A cells by confocal microscopy. Fig. 1b shows that the t-SNAREs (red) are located on the plasma membrane, whereas the v-SNARE (green) is located in intracellular vesicular pools (Bock *et al*. 2001; Koticha *et al*. 2002; Sudhof and Rothman 2009).

Next, we explored a possibility of delivering botulinum proteases, which cleave membrane-embedded SNAREs, into normally resistant N2A cells. As it has been observed that transfection reagents are capable of causing the internalization of the Botulinum A and E proteases targeting SNAP25 (Kuo *et al*. 2010), we tried an array of transfection reagents to deliver the type C and D proteases, which, respectively, target t-SNAREs and the v-SNARE. Fig. 1c and d (upper panels) show that Lipofectamine LTX and FuGENE HD were able to deliver the C and D proteases into N2A cells very efficiently as evidenced by changes observed in immunoblotting of the SNARE proteins. TransPass, a *bona fide* protein transduction reagent, was also efficient, but caused cell death as evidenced by a reduction in total SNAP25 protein in the loading controls at the tested dose (Fig. 1c and d upper panels). We chose to use Lipofectamine LTX for subsequent experiments because of its excellent tolerance by cells. Fig. 1c and d (middle panels) show that a dose of 0.2 μg/mL of botulinum proteases was sufficient to achieve near-complete SNARE cleavage upon 20 h incubation. This dose was used in all subsequent experiments. Fig. 1c (bottom panel) also shows a time course of type C protease activity, where a complete cleavage of the syntaxin substrate can be observed even within 4 h, whereas the SNAP25 cleavage slightly lags behind and is completed within 8 h. We also observed a near-complete cleavage of synaptobrevin by the D protease within 4 h (Fig. 1d, bottom panel). Together, extensive cleavage of all three SNAREs in Lipofectamine LTX-treated cells can be observed in the presence of the respective botulinum proteases at 0.2 μg/mL within 8 h.

### Cytotoxic effects of the botulinum protease serotypes C and D

**Fig. 2:**
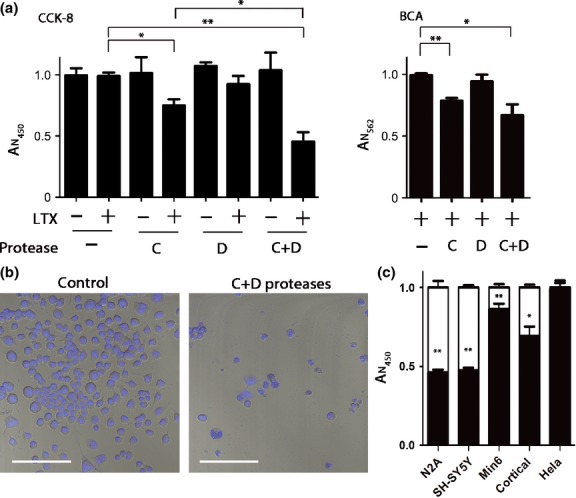
Cytotoxicity of the botulinum C and D proteases. (a) Cell counting assay (CCK-8) was used to monitor neuroblastoma N2A cell viability 40 h after application of the proteases in the presence or absence of Lipofectamine LTX. Botulinum protease type C significantly impaired viability compared with a control condition (*p < 0.01). Application of both proteases with Lipofectamine LTX further lowered cell survival (**p < 0.001). Absorbance at 450 nm (A_N__450_) normalized to control values is proportional to cell count (left panel). The right panel shows the absorbance at 562 nm (A_N562_) proportional to total protein content in the wells as determined by the bicinchoninic acid (BCA) assay. Botulinum protease type C in the presence of Lipofectamine LTX significantly lowered viability compared with control condition (**p < 0.001) while the addition of the type D protease reduced survival further (*p < 0.01). Results are presented ± SD. (b) Confocal images showing a reduction in number of N2A cells stained with Hoechst 33342. Left panel shows cells treated with Lipofectamine LTX alone, whereas right panel shows cells treated with C and D proteases in the presence of Lipofectamine LTX. White bar: 100 μm. (c) CCK-8 assay was used to monitor viability of the indicated cells following the addition of Lipofectamine LTX with or without botulinum proteases. Control conditions where cells were treated with Lipofectamine LTX alone are shown as white columns. Results were normalized to control (LTX alone, ± SD). A significant (**p < 0.001) loss of viability compared with untreated controls was observed in N2A, SH-SY5Y, Min6, and rat brain cortical cells, which comprise both neurons and non-neuronal cells (*p < 0.01).

It is well-known that BoNT/C has neurotoxic properties, but botulinum effects on survival of neuroendocrine cells have not been specifically addressed. To test cell viability of neuroblastoma cells, we used a cell counting kit (CCK-8). Fig. 2a (left panel) shows the normalized signals exhibited by N2A cells following treatment with botulinum proteases. The type C protease significantly reduced cell viability after a 40 h exposure (*p < 0.01). Curiously, this effect was enhanced with the addition of the type D protease, whereas the type D protease alone was not sufficient to significantly affect cell viability. As dehydrogenase activity might be affected by botulinum-induced changes in membrane trafficking, we corroborated our observations using the BCA protein assay which measures total protein content present in the wells which is a direct measure of adherent cells. The protein content of adherent cells (Fig. 2a, right panel) precisely correlated with the CCK-8 kit results (*p < 0.01; **p < 0.001). Lipofectamine LTX on its own did not affect cell viability (Fig. 2a). In further experiments, we chose to study the combined effect of type C and D proteases as together they mediated the most potent cytotoxicity. Fig. 2b shows a confocal image of botulinum-treated cells versus control cells, with a substantial reduction in the number of cells evident following the protease treatment. Next, we tested other cell lines of neuroendocrine origin and which carry syntaxin 1, SNAP25, and synaptobrevin 2 (Fig. 2c). We observed a significant cytotoxic trend in the human neuroblastoma cell line SH-SY5Y (*p < 0.01) and substantial cytotoxic effect in mouse insulinoma Min6 cells (*p < 0.01) and neurons (*p < 0.01) compared with their respective untreated controls (100%). The non-neuroendocrine human cell line Hela did not show loss of survival which can be explained by the low levels of botulinum proteolytic substrates in these cells.

**Fig. 3:**
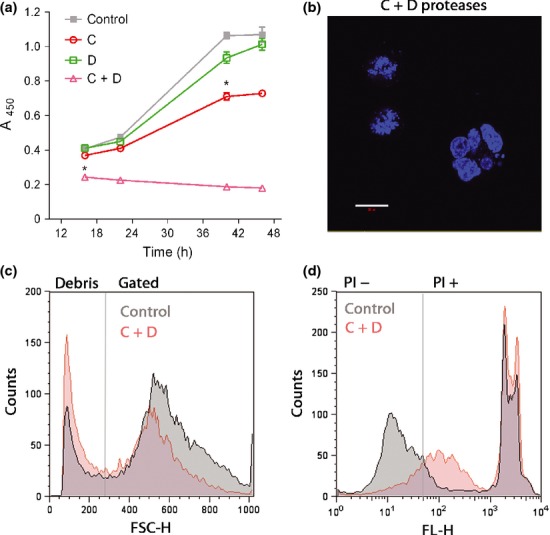
Time course of botulinum-triggered cytotoxic effects. (a) A late onset of toxicity observed following application of type C, type D, and C and D proteases. A significant (*p < 0.01) reduction in cell viability is observed at 16 h post-transduction for the combined C and D protease, whereas a significant (*p < 0.01) reduction in the type C protease-treated cells can be observed after 40 h. Results are presented ± SD. (b) Confocal image showing fragmented nuclei of N2A cells treated with the C and D proteases, indicating apoptosis. White bar: 20 μm. (c) Histogram of the forward scatter height (FSC-H) observed by flow cytometry. The gated region on the right contained morphologically normal (i.e., size and complexity) cells, whereas the left section shows the cell debris. Botulinum protease-treated cells (red) exhibit higher debris count compared with control (gray) (*p* < 0.05). (d) Histogram representation of FL-H (fluorescent height) signal of the propidium iodide (PI)-stained morphologically normal cells indicates a significant increase in cell death following application of botulinum proteases (*p* < 0.01).

We next investigated the time course and features of the N2A cell demise. Fig. 3a shows that the loss in cell viability following botulinum treatments (C and D) continues to occur for at least 40 h. The type C protease showed a significant reduction in viability after 36 h (*p < 0.01), whereas the type C and D protease displayed a significant reduction as early as 16 h (*p < 0.01). When observed under higher magnification (Fig. 3b), the botulinum protease C- and D-treated cells often displayed nuclear abnormalities, as would be expected during programmed cell death (Danial and Korsmeyer 2004). For flow cytometry, cells with normal morphology were gated according to the forward and side scattering patterns using untreated cells as a control and then total percentages of gated cells were calculated (Fig. 3c). There was a significant reduction (*p* < 0.05) in the percentage of morphologically normal cells in the sample cotreated with C and D proteases. Fig. 3d shows the analysis of these gated cells proportional to their PI labeling, a widely used necrosis and late apoptosis marker (Lecoeur 2002). A significant rightward shift in PI-positive cells suggests increased cell death of the C and D proteases-treated cells (*p* < 0.01). We could not rely on the Annexin V-FITC labeling of apoptotic cells as lipofection by itself alters lipid balance in the plasma membrane and thus gives artificially high Annexin V binding without affecting cell survival.

### Botulinum protease-cleaved SNARE fragments mediate cytotoxicity

**Fig. 4:**
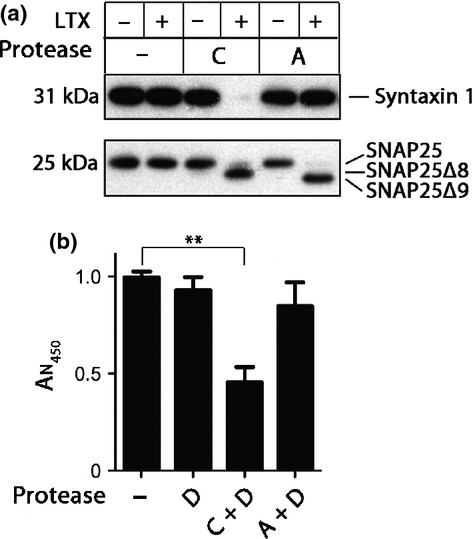
The importance of syntaxin cleavage in botulinum-triggered cell demise. (a) Immunoblot showing that, in the presence of Lipofectamine LTX, the botulinum protease type C cleaves both syntaxin 1 and SNAP25 in N2A cells, whereas the type A protease cleaves SNAP25 only. Type C protease removes the last eight residues of SNAP25 (SNAP25Δ8), whereas type A removes the last nine (SNAP25Δ9). (b) The bar chart showing N2A cell demise following a 40 h treatment with the indicated proteases. Type A-induced cleavage of SNAP25, even in the presence of the type D protease, is insufficient to drive the cytotoxic effects. In contrast, cleavage of syntaxin by type C protease has a strong effect on cell survival (**p < 0.001). Results are normalized to control and presented ± SD.

As botulinum type C protease cleaves both syntaxin and SNAP25, we next aimed to address whether cleavage of syntaxin or SNAP25 is responsible for the cytotoxic effects observed above. We used the BoNT/A protease, the main ingredient of the BOTOX and Dysport preparations, which exclusively cleaves SNAP25. Fig. 4a shows the comparative proteolytic activity of the type A and C proteases as seen in western immunoblotting. The major difference which can be observed between the A and C proteases is the cleavage of syntaxin 1. Fig. 4b shows a significant reduction in cell viability exclusively when the C protease is used together with the type D protease, whereas the D protease alone or the A and D proteases in combination exhibit no discernible cytotoxicity. This result indicates that it is the cleavage of syntaxin by the type C protease that is the driving force for the observed cytotoxicity.

**Fig. 5:**
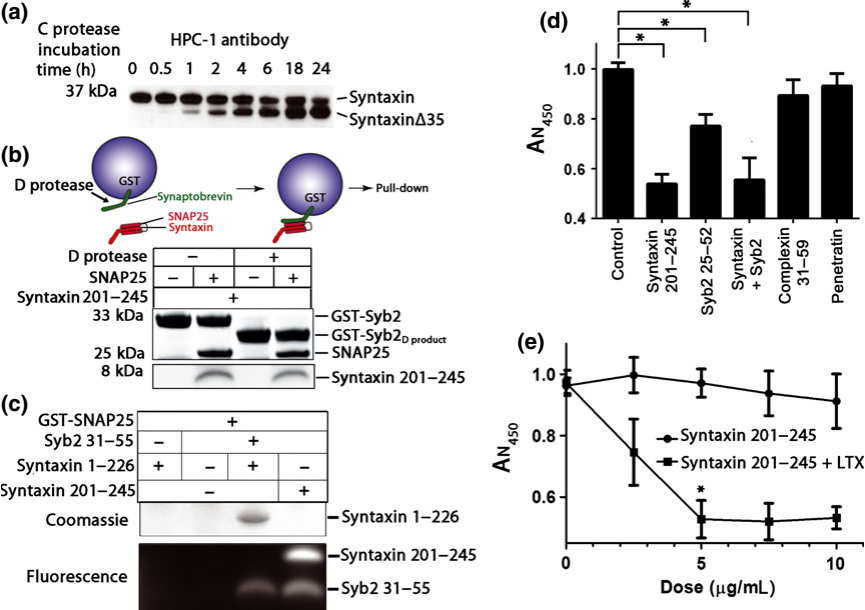
Shorted soluble *N*-ethylmaleimide-sensitive factor attachment protein receptor (SNARE) fragments can interact and cause cytotoxicity upon transduction. (a) Western immunoblotting of N2A cells, treated with 0.002 μg of type C protease for up to 24 h, reveals cleaved syntaxin fragment 1-253 (syntaxinΔ35). In this instance immunoblotting was performed using the HPC-1 anti-syntaxin antibody. (b) Schematic of a SNARE pull-down reaction (upper panel). The lower panel showing cleavage of Glutathione *S*-transferase (GST)-synaptobrevin 2 (GST-Syb2) by type D protease and the ability of this product to bind syntaxin fragment (201-245) and SNAP25 as seen in the Coomassie-stained sodium dodecyl sulfate–polyacrylamide gel electrophoresis (SDS–PAGE) gel. Note SNAP25 is required for the syntaxin pull-down by GST-Syb2 and GST-Syb2_D product_ highlighting the ternary nature of the SNARE interaction. (c) GST-SNAP25 pull-down experiment reveals interaction of the indicated shortened synaptobrevin and syntaxin fragments, strictly in a ternary manner. Syntaxin fragment 1-226 incorporating the regulatory syntaxin head with a truncated SNARE motif was revealed by Coomassie staining, whereas short SNARE peptides, carrying FITC, were imaged in the Bio-Rad XRS imaging station. (d) Bar chart showing cell survival measured using CCK-8 assay. Intracellular delivery of syntaxin fragment (201-245) and synaptobrevin (25-52) peptide using Lipofectamine LTX causes a significant reduction in cell viability, whereas transduction of control peptides, complexin (31-59) and penetratin, at the same concentration does not lead to cell death (*p < 0.05). Results are normalized to control and presented ± SD. (e) The short syntaxin fragment (201-245) applied to N2A cells causes cell death in a dose-dependent manner. Changes in cell viability are shown with and without Lipofectamine LTX. A significant loss of viability can be observed at doses as low as 5 μg/mL (*p < 0.01, ± SD).

Finally, we tested whether transduction of type C protease does result in production of syntaxin fragments, as previous results (Fig. 1) indicated loss of immunoreactivity. We used an antibody directed against the head domain of syntaxin 1 (clone HPC-1) and found a clear appearance of the syntaxin proteolytic fragment within several hours of application of the botulinum protease at a dose of 0.002 μg/mL (Fig. 5a).

As botulinum-cleaved syntaxin and synaptobrevin products are cleaved from their transmembrane anchors, they could be soluble and may potentially form aberrant SNARE complexes. We tested whether short SNARE fragments can form such ternary complexes in bead pull-down assays. We first used GST-synaptobrevin 2 attached to beads. As can be seen in Fig. 5b, the D protease cleaved the bead attached GST-synaptobrevin 2 (22-84) very efficiently generating a cleavage product (GST-Syb2_D product_) still attached to beads through its N-terminal GST fusion. The BoNT/C protease produces the syntaxin 1-253 aa fragment which encompasses the short syntaxin peptide 201-245 (Ferrari *et al*. 2012). Fig. 5b also shows that GST-synaptobrevin, cleaved or not, was able to pull-down both SNAP25 and the short syntaxin fragment. This suggests that botulinum protease C- and D-released products can still interact to form ternary SNARE complexes. Furthermore, we used GST-SNAP25 bound to beads to investigate SNARE interactions with truncated syntaxin 1-226 and a minimal synaptobrevin further reduced to only 25 amino acids. Fig. 5c shows that the short synaptobrevin and syntaxin fragments, with or without the syntaxin regulatory head domain, can still form SNARE complexes evidenced in the pull-down reactions.

To investigate whether shortened SNARE fragments can trigger cytotoxicity, we treated the N2A cells with syntaxin 1 (201-245, 5 μg/mL) and/or synaptobrevin 2 (25-52, 5 μg/mL) peptides, with or without Lipofectamine LTX. Complexin, a SNARE protein that does not directly contribute to the tetrahelical bundle was used as control. Penetratin, a cell-penetrating peptide, was also used as a negative control to eliminate the possibility that non-specific internalization might contribute to cytotoxicity. Fig. 5d shows that N2A cells treated with the shortened SNARE peptides exhibited decreased cell survival compared with control conditions. The syntaxin fragment alone was very efficient even at low concentrations (Fig. 5d and e), thereby masking possible cumulative effects. No decrease in viability was observed with the same concentrations of complexin or penetratin demonstrating the importance of the SNARE ternary interactions involving syntaxin as the major driving factor in triggering cytotoxicity.

## Discussion

Botulinum neurotoxins have been invaluable in the studies of the SNARE machinery. Here, we demonstrate a straightforward and easy method to deliver botulinum type D and C proteases into normally refractory cells to cleave membrane-embedded v- and t-SNAREs. Our results expand upon previous observations of the delivery of SNAP25 proteases type A and E (Kuo *et al*. 2010). A number of studies highlighted BoNT/C cytotoxic effects on central and peripheral neurons, (Foran *et al*. 2003; Berliocchi *et al*. 2005; Zhao *et al*. 2010; Peng *et al*. 2013), but possible cytotoxic effects in normally resistant neuroendocrine cells have not been addressed. Our newly discovered ability to introduce botulinum proteases in all cultured cells suddenly revealed cytotoxic effects especially for the type C botulinum protease. We observed increase in nuclear PI labeling and nuclear abnormalities following botulinum type C and D protease application which correspond to necrosis and late apoptosis, respectively. This is consistent with the observed caspase 3 activation and apoptosis of cerebellar neurons upon BoNT/C administration (Berliocchi *et al*. 2005). Our data show that botulinum protease-induced cell death can occur independent of axonal changes.

The precise mechanisms of botulinum cytotoxicity in neurons are not fully understood. As one possible mechanism which warrants further investigations, we propose that botulinum cleavage products that still contain SNARE interacting domains are able to compete with normal SNARE interplay and disrupt functionally important trafficking and vesicular fusion events. Our results showing synergistic effects of botulinum proteases on cytotoxicity of non-neuronal cells, as well ternary complex formation by shortened SNARE peptides, suggest that cellular well-being generally relies on proper SNARE localization and precisely regulated stoichiometry of SNARE interactions. Although a recent study suggested that the general presence of neuronal SNARE proteins is important for neuronal survival, this can be explained by the importance of intercellular signaling for the maintenance of neuronal circuitry (Peng *et al*. 2013). Similarly, it could be argued that cell–cell communication is important for cancer cell survival. However, it is well-known that cancer neuroendocrine cells sometimes lose secretory pathway components and yet they are able to grow and proliferate in a normal way (Pance *et al*. 2006). As botulinum-released SNARE fragments can form ternary complexes they may contribute to deregulation of precise membrane trafficking necessary for correct cell function (D'Alessandro and Meldolesi 2013).

Whereas botulinum type C protease possesses the most potent cytotoxic effects, our data show that it is specifically cleavage of syntaxin rather than SNAP25 which mediates cell toxicity. Indeed, when we tested the type A protease, which only cleaves SNAP25, no cytotoxic effects were observed. A previous study suggested that BoNT/C-cleaved syntaxin degrades too quickly to interfere with intracellular functions, but this inference was based on loss of immunoreactivity (Foran *et al*. 2003). When we probed neuroblastoma cells using a well-known syntaxin 1 antibody we observed a persistent accumulation of the syntaxin fragment triggered by the type C protease treatment which is in accord with a previous study (Tsukamoto *et al*. 2012); evidently the fragments are not completely degraded and may take part in aberrant SNARE interactions. We now show that syntaxin fragments, as short as 45 aa, can drive SNARE complex formation as evidenced by pull-downs with SNAP25 and synaptobrevin, even if the latter cleaved by the botulinum type D protease.

This study raises several questions for future investigations. The mechanism of Lipofectamine LTX-induced transduction of botulinum enzymes and small peptides remains unclear because of proprietary reasons and the active ingredients should be understood considering that this reagent was more effective than *bona fide* protein transduction preparations. It is also not clear, why generation of syntaxin fragments is more cytotoxic compared with synaptobrevin despite that in both cases botulinum proteases sever the SNARE membrane-embedded parts (Peng *et al*. 2013). It will be revealing to detect even smaller SNARE degradation products following botulinum treatment as small syntaxin and synaptobrevin fragments can still form irreversible SNARE complexes (Ferrari *et al*. 2012). This is also important in view of SNARE degradation and deregulation which apparently takes place in neurodegenerative disorders (Garcia-Reitbock *et al*. 2010). The importance of normal SNARE function was previously suggested by a genetic study where deletion of synaptic vesicle protein cystein string protein α, a SNARE chaperon, led to massive neurodegeneration *in vivo* (Sharma *et al*. 2011).

Our results have implications for the use of BoNT/C in biomedical applications and further validate the safer cytological characteristics of BoNT/A-based preparations such as BOTOX and Dysport. BoNT proteases are currently of high pharmaceutical interest (Chaddock and Marks 2006; Chen and Barbieri 2009; Foster and Chaddock 2010; Davletov *et al*. 2012; Ferrari *et al*. 2013; Naumann *et al*. 2013) and the newly highlighted delivery mechanisms could greatly advance the ongoing botulinum research. Considering the observed cytotoxicity, the botulinum proteases could be utilized as molecular surgery tools for neuroendocrine cancer therapies. Targeted delivery of botulinum proteases could even be combined with short syntaxin peptides for cumulative cytotoxic effects. Many neuroendocrine tumors give rise to complicated endocrinopathies that require constant monitoring and problematic surgical interventions (Bangaru *et al*. 2010; Batcher *et al*. 2011). The possibility of using certain botulinum proteases/SNARE peptides to destroy cancerous neuroendocrine cells paves the way for devising strategies to both block the pathological release of hormones and concomitantly halt tumor proliferation.

## Abbreviations

BCA: bicinchoninic acid
BoNT: botulinum neurotoxins
BSA: bovine serum albumin
CCK-8: cell counting kit 8
CSPα: cystein string protein α
DMEM: Dulbecco's modified eagle medium
FCS: fetal calf serum
N2A: mouse neuroblastoma 2A (Neuro2A)
PBS: phosphate-buffered saline
PI: propidium iodide
SNAP25: synaptosomal-associated protein of 25 kDa
SNARE: soluble *N*-ethylmaleimide sensitive factor attachment protein receptor
Syb: synaptobrevin
